# Evaluation of *K-ras* and *p53* expression in pancreatic adenocarcinoma using the cancer genome atlas

**DOI:** 10.1371/journal.pone.0181532

**Published:** 2017-07-25

**Authors:** Liming Lu, Jingchun Zeng

**Affiliations:** 1 The Second Affiliated Hospital of Guangzhou University of Chinese Medicine, Guangdong Provincial Hospital of Chinese Medicine, Guangzhou, China; 2 The First Affiliated Hospital of Guangzhou University of Chinese Medicine, Guangzhou, China; Virginia Commonwealth University, UNITED STATES

## Abstract

Genetic alterations in *K-ras* and *p53* are thought to be critical in pancreatic cancer development and progression. However, *K-ras* and *p53* expression in pancreatic adenocarcinoma have not been systematically examined in The Cancer Genome Atlas (TCGA) Data Portal. Information regarding *K-ras* and *p53* alterations, mRNA expression data, and protein/protein phosphorylation abundance was retrieved from The Cancer Genome Atlas (TCGA) databases, and analyses were performed by the cBioPortal for Cancer Genomics. The mutual exclusivity analysis showed that events in *K-ras* and *p53* were likely to co-occur in pancreatic adenocarcinoma (Log odds ratio = 1.599, *P* = 0.006). The graphical summary of the mutations showed that there were hotspots for protein activation. In the network analysis, no solid association between *K-ras* and *p53* was observed in pancreatic adenocarcinoma. In the survival analysis, neither *K-ras* nor *p53* were associated with both survival events. As in the data mining study in the TCGA databases, our study provides a new perspective to understand the genetic features of *K-ras* and *p53* in pancreatic adenocarcinoma.

## Introduction

Pancreatic cancer (PC) happens when pancreatic cells start to proliferate without control and form a mass. As the most frequent type of PC, pancreatic adenocarcinoma takes the largest proportion 85% of all cases, which is usually equivalent to the expression "pancreatic cancer". The global annual incidence rate for PC is about 8/100,000 persons.[1] Recently it is reported that the treatment is promising in the near future.[2] Furthermore, as the overall 5-year survival rate was only 5%, PC remains one of the most lethal cancers [3,4]. Meanwhile, an overall reduction in cancer-related mortality has occurred among lung, breast, colorectal and prostate cancer over the last few decades.[5]

Nowadays, how to improve the prevention and treatment of pancreatic adenocarcinoma remains a critical issue. Knowing PC’s molecular biology benefits the development of new approaches to advances in its clinical management. The occurrence and evolution of pancreatic adenocarcinoma involves multiple genetic alterations. Genetic alterations *K-ras* and *p53* are considered key to both pancreatic cancer progression.[6] The *K-ras* proto-oncogene encodes a small 21 kDa protein (p21ras). This protein possesses the activity of GTPase, enabling inactivation of cancer cell.[7] It is common that genetic deletions or mutations of the *p53* tumor suppressor gene exists in pancreatic adenocarcinoma (40–87% of cases). Activation of the *p53* tumor suppressor gene causes cell cycle arrest by encoding a nuclear phosphoprotein and binding directly to DNA.[8]

Previous studies tried to explore the correlation of these genes’ alterations and clinical features and survival data.[9–19] The conclusions on *K-ras*, *p53* mutations and prognosis is inconsistent. Furthermore, few studies have explored the relationship of mutations in *K-ras* and *p53* together. It is important for researchers to make it clear that *K-ras* and *p53* are on different pathways, or are part of a common pathway of inactivation of pancreatic adenocarcinoma. The Cancer Genome Atlas (TCGA) Data Portal contains information on DNA, RNA, proteins and survival status in various cancers.[20,21] However, the relationship between *K-ras* and *p53* expression in pancreatic adenocarcinoma was not clear in genes’ alterations and clinical outcomes. This study aimed to assess the genetic alterations of *K-ras* and *p53* and their relationship in pancreatic adenocarcinoma in TCGA data sets. Additionally, we correlate these changes with clinical outcomes.

## Materials and methods

### Gene expression databases

Information regarding *K-ras* and *p53* alterations, mRNA expression, and protein in pancreatic adenocarcinoma can be obtained from The Cancer Genome Atlas (TCGA) database, an open access database publicly available at http://www.cbioportal.org.[21,22]

Before visualizing and analyzing genomic alterations of *K-ras* and *p53* in the TCGA data on pancreatic adenocarcinoma, we selected several options in the web interface of cBioPortal. We selected cancer study “pancreatic adenocarcinoma (TCGA)” and data type priority “Mutation and CNA (DNA copy-number alterations).” For the gene set of interest, terms of “*K-ras p53*” were entered into the input box. No statements of approval or informed consent were required for our study as we obtained data from an open access database.

### Genomic alterations summary

An OncoPrint was used to summarize the genomic alterations of *K-ras* and *p53* through tumor samples. On the table, rows represented genes and columns represented samples. Genomic alterations including mutations, CNA (amplifications and homozygous deletions), and changes in gene expression were summarized by glyphs and color coding. This was a preliminary way to know about the different gene signaling in pancreatic adenocarcinoma. In this section, mutual exclusivity and co-occurrence between *K-ras* and *p53* were analyzed. In mutually exclusive, gene-related events associated with a particular cancer are often mutually exclusive in a group of tumors—that is, only one genetic event is likely to exist in each cancer sample. The other situation is the co-occurrence that multiple genes are altered in the same cancer sample.[21] This was a preliminary way to gather information about the different gene signaling in pancreatic adenocarcinoma.

### Mutations in *K-ras* and *p53* in pancreatic adenocarcinoma

From the Mutations tab, the location and frequency of all mutations in Pfam protein domains were given. The gray bars meant the whole lengths of the *K-ras* and *p53* proteins and the bottom of each gray bar displayed the number of amino acids. Protein domains were showed by the green, red, and blue boxes. The locations and frequencies of genes were showed by the lines and dots. Red represented nonsense or frameshift mutations, green represented missense mutations, and black represented in-frame deletions.[23]

### Network analysis

The network includes all neighbors of *K-ras* and *p53*.[21] Only neighbor genes with the highest alteration frequency (only the 50 neighbors were presented if more than 50 neighbor genes existed) in addition to the query genes were shown. Color coded edges were used to highlight the frequency of gene alteration.

### Survival analysis

From survival analysis, overall survival and disease-free survival differences were compared between samples with more than or equal to one alteration of query gene(s) and samples without alteration. This was done if the survival data were available.

### Statistics

For correlation analysis, a scatter plot of mRNA expression versus copy-number status or the protein level versus mRNA option in each sample was presented. For survival analysis, Kaplan-Meier plots with a logrank test were performed to compare the overall survival and the disease-free survival of pancreatic adenocarcinoma with at least one alteration or without alteration in query gene(s). Samples with over-expression were identified by a threshold of Z>2 (mean expression over 2 SDs). The *a* level was set at 0.05. All of the analyses mentioned above were performed in cBioPortal. For the details of original data, see S1 and S2 Datasets.

## Results

### Genomic alterations summary

**Fig 1:**
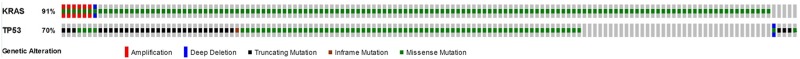
The OncoPrint tab.

From the OncoPrint (Fig 1), 140 (94%) out of 149 cases had an alteration in no less than one of the two genes. Specifically, 91% cases had an alteration in *K-ras*, most of which were missense mutations. Others included a few amplifications and deep deletions. 70% of the cases had an alteration in *p53*, consisting mainly of missense mutation and truncating mutation. The mutual exclusivity analysis showed that events in *K-ras* and *p53* were likely to co-occur in pancreatic adenocarcinoma (Log odds ratio = 1.599, *P* = 0.006).

This result illustrated that the gene signaling in pancreatic adenocarcinoma was mediated by the activation of *K-ras* through missense mutations, or by the inactivation of *p53* through truncating mutation and missense mutation.

### Mutations in *K-ras* and *p53* in pancreatic adenocarcinoma

**Fig 2:**
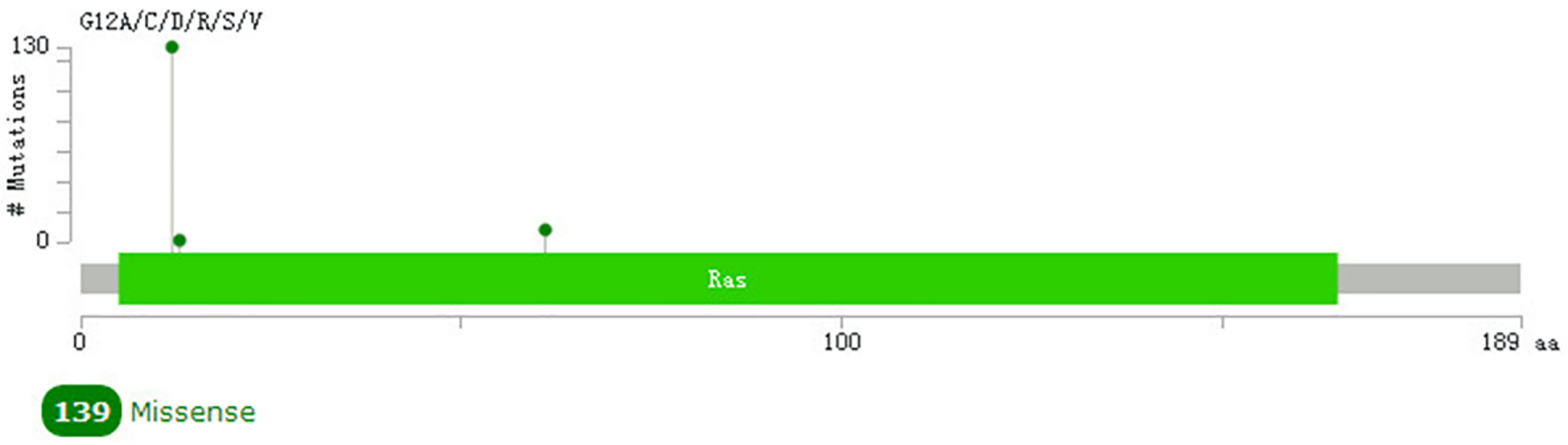
Mutation diagram of *K-ras* in pancreatic adenocarcinoma.

**Fig 3:**
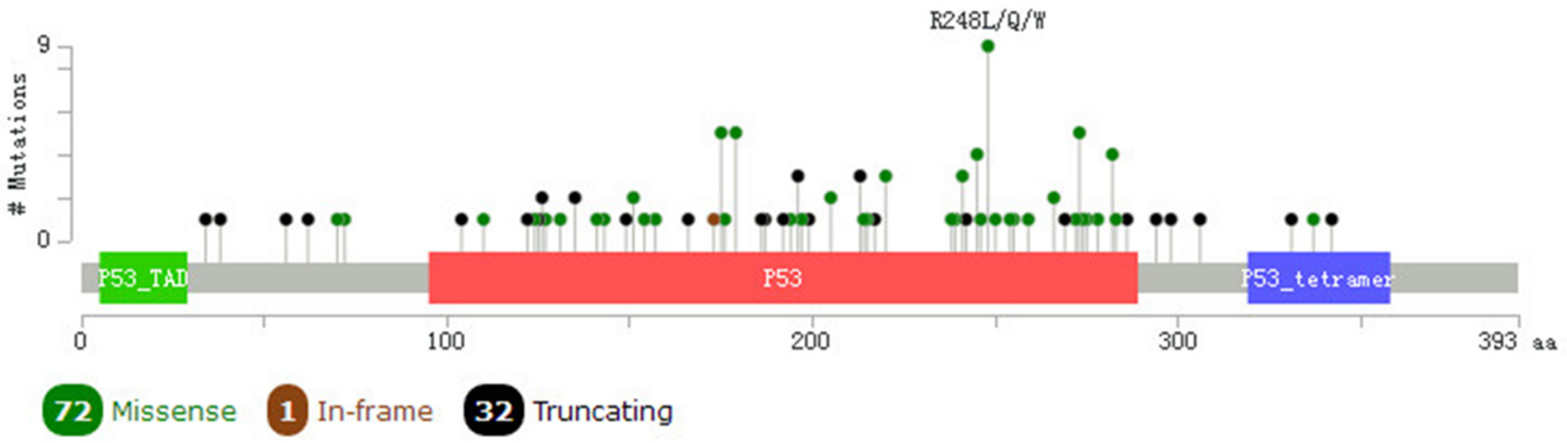
Mutation diagram of *p53* in pancreatic adenocarcinoma.

The graphical summary of the mutations showed that there were 139 *K-ras* nonsynonymous mutations in pancreatic adenocarcinoma samples, and 130 of them were G12C/D/R/S/V in the kinase domain (Fig 2). There were 105 *P53* nonsynonymous mutations in pancreatic adenocarcinoma samples, 9 of them being R248L/Q/W in the kinase domain (Fig 3), illustrating that these were hotspots for protein activation.

### Network analysis

**Fig 4:**
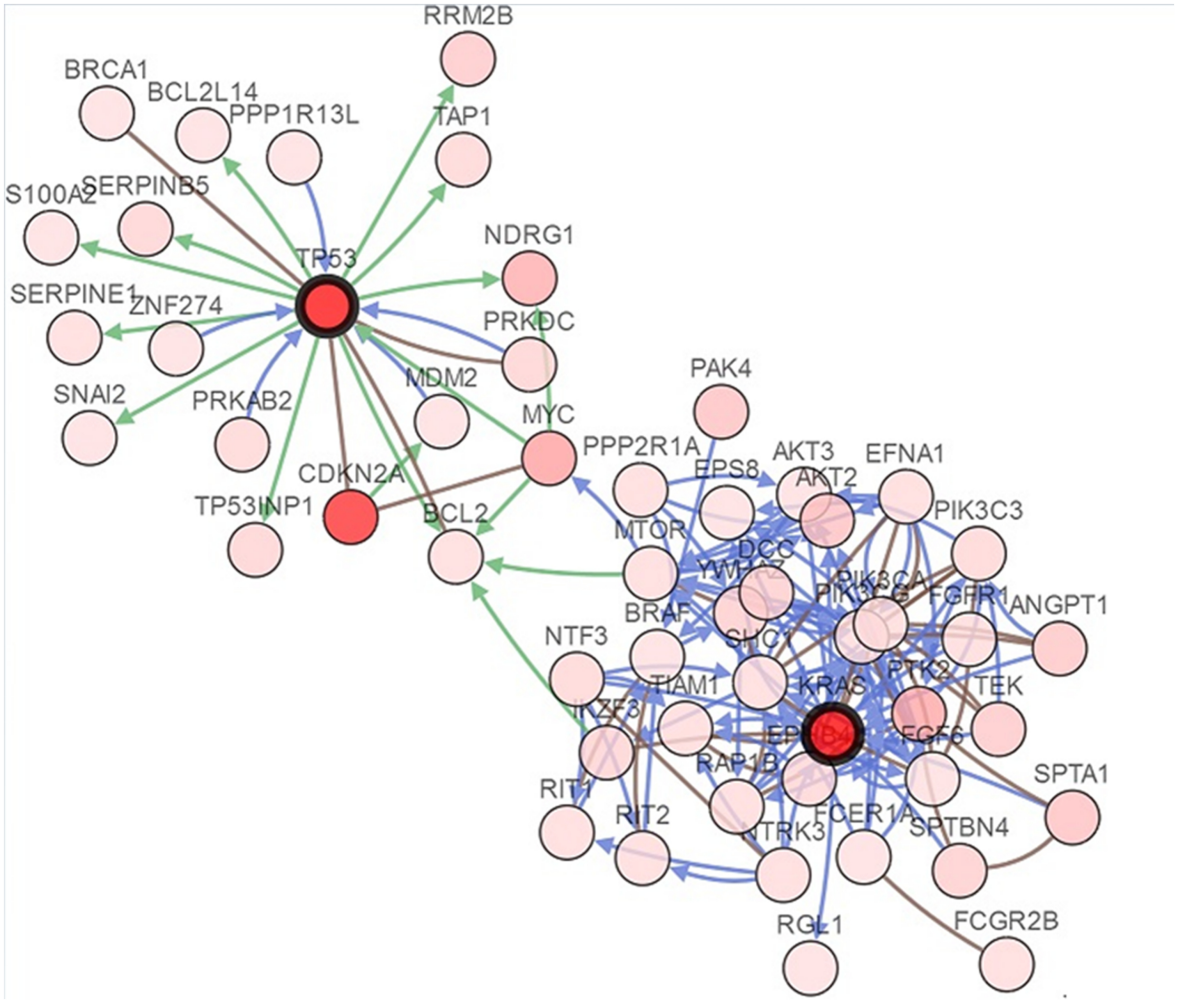
Network analysis of the *K-ras* and *p53* neighborhood in pancreatic adenocarcinoma.

Network view of the *K-ras* and *p53* neighborhood in pancreatic adenocarcinoma was presented in Fig 4. The query genes, *K-ras* and *p53* were depicted with a thick border and neighbor genes were distributing around them. Interestingly, no solid association between *K-ras* and *p53* was observed in pancreatic adenocarcinoma.

### Survival analysis

**Fig 5:**
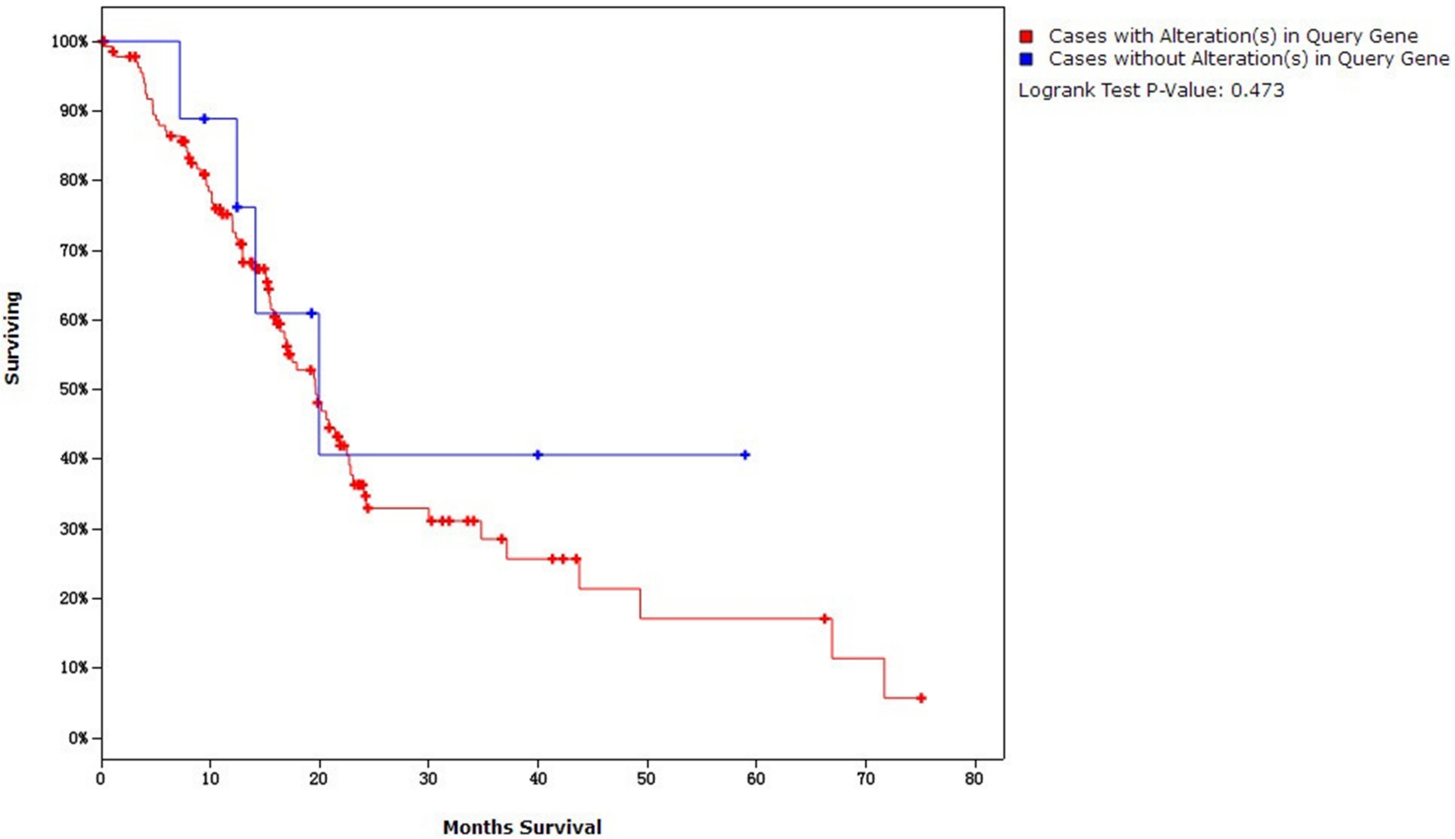
Overall survival analysis.

**Fig 6:**
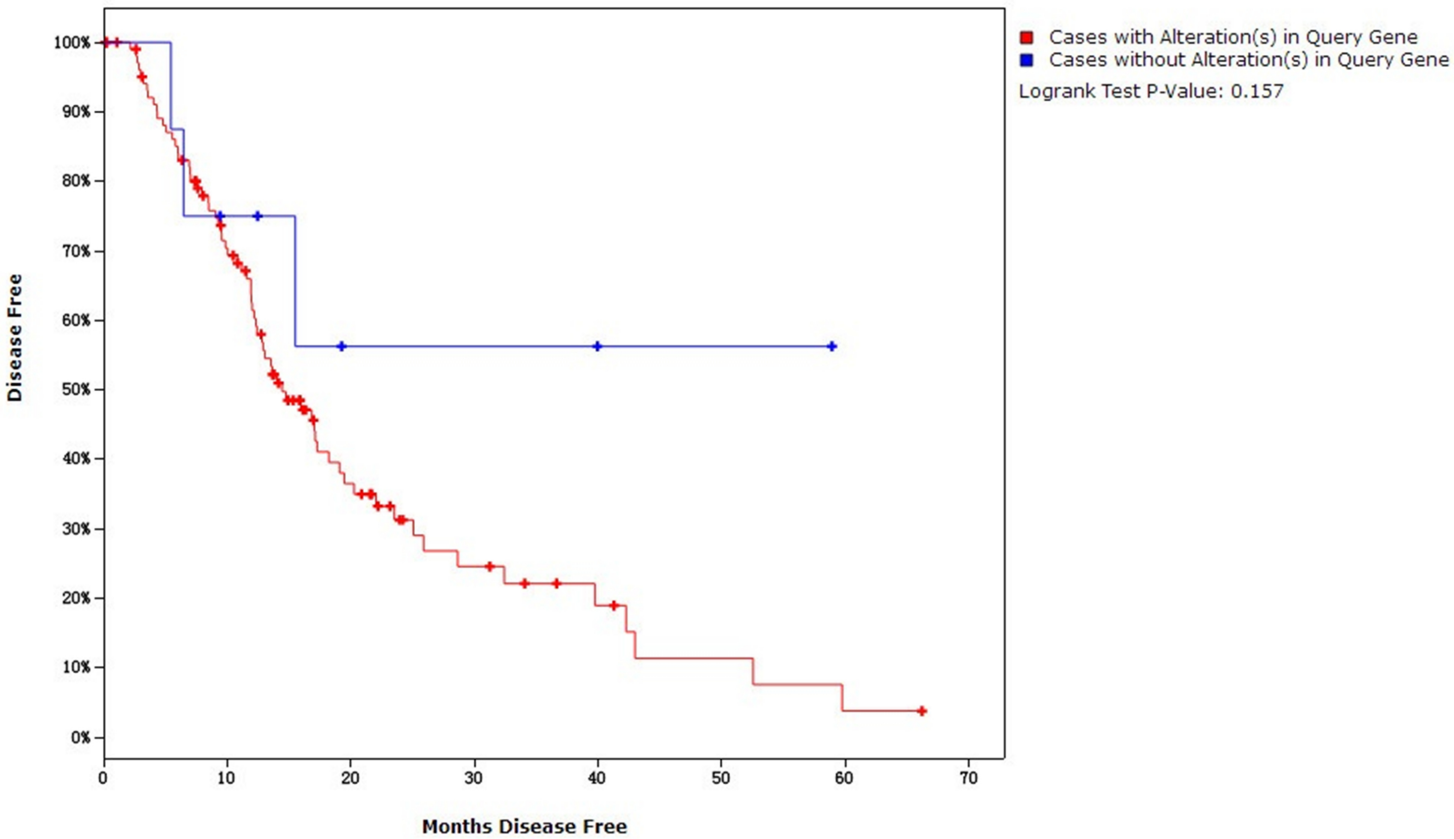
Disease-free survival analysis.

Kaplan-Meier plots was used to compare overall survival and disease free survival in pancreatic adenocarcinoma cases with or without *K-ras* and *p53* over-expression. For the overall survival analysis, mutations simultaneously in *p53* and *K-ras* were found in 54.73% (81/148) of cases and were not related to decreased overall survival (19.65 months versus 19.94 months for those without both mutations, *P* = 0.473) (Fig 5). For the disease free survival analysis, mutations in *K-ras* and *p53* were simultaneously found in 61.74% (71/115) of cases and were not associated with decreased disease free survival (14.45 months versus 16.75 months for those without both mutations, *P* = 0.157) (Fig 6). Similarly, neither *K-ras* nor *p53* were associated with both survival events (Figures not presented).

## Discussion

In the current study, we have used The cBioPortal for Cancer Genomics as a tool for exploring, visualizing, and analyzing the biological and clinical features of *p53* and *K-ras* alterations in pancreatic adenocarcinoma cases from TCGA databases. As far as we know, this is the first data mining study to explore the relationship between alterations of *K-ras* and *p53* and patient prognosis in TCGA databases.

Similar to previous reports, *K-ras* has high alteration frequency of 47–100% in pancreatic adenocarcinoma.[24–26] *K-ras* alterations are also found in benign pancreatic disease and at all stages of pancreatic ductal anaplasia.[27] *K-ras* alterations are thought to be an early event in pancreatic carcinogenesis. *p53* has the function of enhancing G1 arrest for DNA damage and reducing damaged DNA to be replicated. In our study, *p53* alterations were found in 70% cases of pancreatic adenocarcinoma, a proportion similar to previous literature which has reported a range of 40–87%.[28,29]

Mutual exclusivity is inferred by a statistical analysis which can reveal a rough relationship between different genes. The network analysis can tell us more about the mechanisms of interaction among the different genes. Our study observed that *K-ras* and *p53* were likely to co-occur, and that network analysis found no solid association between them in pancreatic adenocarcinoma. “No solid association” does not mean no association. It can be seen from Fig 4 that *K-ras* and *p53* have some association, but that this association is “no solid” when compared with the other 48 neighbor genes. This suggests that *K-ras* and *p53* alterations mostly coexist in pancreatic adenocarcinoma, but alterations in these genes are on independent pathways to pancreatic adenocarcinoma and are not in a common way of cumulative gene variation. This is consistent with a previous cohort study.[8] This data mining study cannot draw an initiating cause of pancreatic adenocarcinoma, as only prospective experimental studies can confirm this hypothesis. This is one limitation for our study.

For survival analysis, no association was observed between the two genes and survival events in our study. Coincidentally, most papers published in recent years have failed to prove the association of these two genes’ molecular changes and patient prognosis. Kawesha et al.[30] found that *K-ras* mutation alone was not related with survival, but significant differences in survival might exist due to the type of *K-ras* mutation. Shin et al.[6] verified that *K-ras* mutation alone was related with patients’ survival, and that GAT subtype had the closest relationship with survival among the Korean population. These findings suggest that *K-ras* mutation has a different prognosis value of pancreatic adenocarcinoma in different geographic locations and populations. Additionally, the majority of recent published studies have reported no association between *p53* alterations and patient survival,[31–34] while some other studies have shown that mutations of p53 gene could reduce postoperative survival.[35,36] From our results, mutations in *K-ras* and *p53* were not related with decreased overall survival and disease-free survival. This was due to a small sample size which was not sufficient to confirm explanatory power.

## Conclusions

In the current study, we used The cBioPortal for Cancer Genomics as a tool for exploring, visualizing, and analyzing the biological and clinical features of *p53* and *K-ras* alterations in pancreatic adenocarcinoma cases from TCGA databases. As far as we know, this is the first data mining study to explore the relationship between alterations of *K-ras* and *p53* and patient prognosis in TCGA databases. Many findings in our research are consistent with previous reports. Interestingly, our study observed that *K-ras* and *p53* alterations mostly coexist in pancreatic adenocarcinoma. Alterations in these genes are on independent pathways to pancreatic adenocarcinoma and are not in a common way of cumulative gene variation. Though neither *K-ras* nor *p53* were associated with both survival events (overall survival and disease free survival) in our study, it provides us a new perspective to simultaneously perform the analysis of genetic alterations and clinical features via data mining.

## Supporting information

S1 Dataset Mutations. (TXT)

S2 Dataset Putative copy-number alterations from GISTIC. (TXT)
